# Supplementary material for: Rapid turnover of life-cycle-related genes in the brown algae
Source: Genome Biol. 2019 Feb 14;20:35. doi: 10.1186/s13059-019-1630-6 (PMC6374913; doi:10.1186/s13059-019-1630-6)
Supplement: Supplementary file 3 — Protein alignments, corresponding Gblocks html file and CDS sequences for the lineage conserved gametophyte-biased genes. (ZIP 2109 kb) [file 13059_2019_1630_MOESM3_ESM.zip › Con_GA_alignments/Ec-19_002810.aa_ali.fasta-gb.htm]

Ec-19\_002810.aa.fasta.trans.aa\_ali.fasta


## Gblocks 0.91b Results

Processed file: **Ec-19\_002810.aa.fasta.trans.aa\_ali.fasta**  
Number of sequences: **2**  
Alignment assumed to be: **Protein**  
New number of positions: **124** (selected positions are underlined in blue)

```
                         10        20        30        40        50        60
                 =========+=========+=========+=========+=========+=========+
Ec-19_002810     MIGHWQFISQRVQAFHLQLASELLRAPSLASKTPTEMDRIRTSFEHACRRSISRIQLGND
MSTRG.41800.1    ------------------------------------------------------------
                                                                             


                         70        80        90       100       110       120
                 =========+=========+=========+=========+=========+=========+
Ec-19_002810     PDLALGNSAERMFSGIVRLHKCSIHQELKEEFGPAVETLLIFVAVDSVSKHALQSFKGAF
MSTRG.41800.1    ------------------------------------------------------------
                                                                             


                        130       140       150       160       170       180
                 =========+=========+=========+=========+=========+=========+
Ec-19_002810     SARAEINRQASAADKPRRKFLPSAAAVAAAAAAAAATATATNLEAERLMAYQNHQQQRLR
MSTRG.41800.1    ------------------------------------------------------------
                                                                             


                        190       200       210       220       230       240
                 =========+=========+=========+=========+=========+=========+
Ec-19_002810     KELQHHHHHRHQSRPGVVRKAAAAQRAAVVGSKSRRATASPSKAKAAKAPRLCRTKSAGS
MSTRG.41800.1    ------------------------------------------------------------
                                                                             


                        250       260       270       280       290       300
                 =========+=========+=========+=========+=========+=========+
Ec-19_002810     SGHGSGGSSSGGSTPRGTGRYRGAGRGGGWNTARLPEEDRNGLLKLPWDGLDAVSEQVKL
MSTRG.41800.1    ------------------------------------------------------------
                                                                             


                        310       320       330       340       350       360
                 =========+=========+=========+=========+=========+=========+
Ec-19_002810     VTDARVQLIEERRLRPSYVPPLSPRDYQYSSGTGGDSTEEPGSPQGHDDSEEAEEDAEAA
MSTRG.41800.1    ------------------------------------------------------------
                                                                             


                        370       380       390       400       410       420
                 =========+=========+=========+=========+=========+=========+
Ec-19_002810     ARRREKQPWFARNVVDHRSAWCGLRNAGARAGLRKVLQPRPWGDPLAPSGGGQEAANSQV
MSTRG.41800.1    ------QPWFVRNVVDHRSAWCGLRDAGERAGLREVLHPRPWGDLRDPSSGARQATQSQV
                       ######################################################


                        430       440       450       460       470       480
                 =========+=========+=========+=========+=========+=========+
Ec-19_002810     LELISAATQAYLKRVLTELSVVAYQRRGGLSEELLQGLEERMEDP-RSPGSALVLYEGLV
MSTRG.41800.1    LELLSSATQSYLARVLKQLVTVAWQRRGGLSDDLLRGVEAQMEDPGLSSSPAVALYSALV
                 #############################################  #############


                        490       500       510       520       530       540
                 =========+=========+=========+=========+=========+=========+
Ec-19_002810     PRFPRYESGGSSTTITTGTPAAAVATTTEDPREVLRREEGAHRRSCLEREAGWEARVAEV
MSTRG.41800.1    P------------------------TIHDDDR------------ACFP-----------A
                 #                                                           


                        550       560       570       580       590       600
                 =========+=========+=========+=========+=========+=========+
Ec-19_002810     MAAADGGRKSRNKLRSFRNAPSQRVGVDSLSAWSESGWLSREQVAANEFARRVGFKRGVG
MSTRG.41800.1    CAAAGTXGNGRN--------------------------------DAVEDADKVG------
                  ###########                                                


                        610       620       630       640       650       660
                 =========+=========+=========+=========+=========+=========+
Ec-19_002810     LSMPASAEWRRKKRMAEAAEARGRGGGGGGGSGGRYAMGGSGQQQQQQQEEGEDQMEIEE
MSTRG.41800.1    ----------------------GRSGGGVGGA----------------------------
                                                                             


                        670       680       690       700       710       720
                 =========+=========+=========+=========+=========+=========+
Ec-19_002810     CDESRLSVDGGGIDARPSVVGVRDEGVGERRKRQSTGNNMDDQSDDDGNRKSRSGSGEDE
MSTRG.41800.1    ------------------------------------------------------------
                                                                             


                        730       740       750       760       770       780
                 =========+=========+=========+=========+=========+=========+
Ec-19_002810     QARALVRDVRRRFHRFGGTTPAAPGGRIEINNADLTEHLRAVMPTCSRESWAILSGLKAR
MSTRG.41800.1    ------------------------------------------------------------
                                                                             


                        790
                 =========+===
Ec-19_002810     LASQRRRAADAGR
MSTRG.41800.1    ------------R
```

```
Parameters used
Minimum Number Of Sequences For A Conserved Position: 2
Minimum Number Of Sequences For A Flanking Position: 2
Maximum Number Of Contiguous Nonconserved Positions: 8
Minimum Length Of A Block: 10
Allowed Gap Positions: None
Use Similarity Matrices: Yes
```

```
Flank positions of the 3 selected block(s)
Flanks: [367  465]  [468  481]  [542  552]  

New number of positions in Ec-19_002810.aa.fasta.trans.aa_ali.fasta-gb:  124  (15% of the original 793 positions)
```
